# International Publication Trends of Studies Comparing Total Ankle Arthroplasty and Ankle Arthrodesis: A Systematic Literature Review

**DOI:** 10.5435/JAAOSGlobal-D-24-00181

**Published:** 2026-01-14

**Authors:** Per-Henrik Randsborg, Taylor Lawson, Hongying Jiang

**Affiliations:** From the Department of Orthopedic Surgery, Akershus University Hospital, Norway (Dr. Randsborg); the Faculty of Medicine, campus Ahus, University of Oslo, Norway (Dr. Randsborg); and the Office of Orthopedic Devices (OHT6), Office of Product Evaluation and Quality at the Center for Devices and Radiological Health, US Food and Drug Administration (FDA), Silver Spring, MD (Dr. Lawson and Dr. Jiang).

## Abstract

**Background:**

End-stage ankle osteoarthritis can be treated surgically by either total ankle arthroplasty (TAA) or ankle arthrodesis (AA). The purpose of this systematic literature review is to analyze international publication trends of comparative studies of TAA and AA.

**Methods:**

A systematic literature review was conducted, searching Embase and PubMed for studies comparing the utilization of TAA and AA published between 1 January, 2010, and the search date (March 18, 2022).

**Results:**

Twenty-one publications comparing TAA and AA were included in the literature review, capturing 68,893 TAA procedures and 206,437 AA procedures in seven different countries or regions. Nine studies originated from the United States, and 12 studies from outside the United States (OUS). The number of studies comparing the utilization of TAA and AA declines sharply after 2013. From 2000 to 2013, the volume of TAAs reported increased globally; however, the number of AA procedures reported initially increased globally but declined dramatically in the United States after 2011, while remained steady in OUS.

**Conclusion:**

The number of studies comparing TAA and AA increased in the early 2000s but has decreased for the past 10 years. The available 30-year literature indicates that the interest for TAA procedures has increased in the United States and some Asian countries, while interest for AAs has declined. In Europe, the reported utilization trend of TAA is generally decreasing.

Ankle arthrodesis (AA) is established as the preferred surgical treatment for end-stage ankle osteoarthritis (OA). Total ankle arthroplasty (TAA) was introduced in the 1970s as an alternative to AA to preserve motion of the ankle joint.^1^ Although the indications of the two procedures are not identical, there is a substantial overlap, and both procedures are indicated for end-stage ankle OA.^2^ The real-world utilization of TAA and AA for ankle OA is not readily available because the arthroplasty registers do not report AA procedures. This systematic literature review was designed to identify, characterize, and compare the publication trends of comparative studies of TAA and AA for the surgical treatment of end-stage ankle OA.

## Methods

### Literature Search

We searched Embase and PubMed for studies published from January 1, 2010, to March 18, 2022, using the medical subject headings terms “ankle,” “arthroplasty,” “replacement,” “arthrodesis,” “fusion,” “incidence,” “utilization,” and “trends.” Articles reporting utilization volume (ie, number of procedures) for adult patients who underwent a TAA or AA procedure for ankle OA were included in the literature review (Table 1). The evidence synthesis followed established methodology for systematic reviews.^3^

**Table 1:** Literature Search Terms, Phrases, and Strategy of PubMed Conducted on March 18, 2022 (The Same Terms and Phrases Were Searched in EMBASE—Details Ignored for Brevity)

No.	PubMed: Query March 18, 2022	Results
#9	#7 NOT #8	1002
#8	(“editorial” [Publication type]) OR (“letter” [Publication type]) OR (“case reports” [Publication type]) OR (“comment” [Publication type]) OR (“guideline” [Publication type])	4,111,597
#7	#5 AND #6	1048
#6	English [Language]) AND ((“2010” [Date—publication]: “3000” [Date—publication])	13,308,372
#5	#3 NOT #4	1503
#4	“Hip” OR “knee” OR “knee arthroplasty” OR “hip arthroplasty” OR “renal” OR “toe” OR “toe joint” OR “elbow” OR “shoulder” OR “hand”	1,722,413
#3	#1 AND #2	2236
#2	“Utilization” OR “trends” OR “rates” OR “volume” OR “health care utilization” OR “incidence” OR “epidemiology”	6,827,132
#1	(“ankle”/exp OR ankle) AND (“arthroplasty”/exp OR “arthroplasty” OR “replacement arthroplasty”/exp OR “replacement arthroplasty” OR “joint prosthesis”/exp OR “joint prosthesis” OR “replacement”/exp OR “replacement” OR “total joint replacement”/exp OR “total joint replacement” OR “scandinavian total ankle replacement”/exp OR “scandinavian total ankle replacement” OR “total ankle prosthesis”/exp OR “total ankle prosthesis” OR “fixed bearing total ankle arthroplasty” OR “mobile bearing prosthesis”/exp OR “mobile bearing prosthesis” OR “fixed bearing prosthesis” OR “arthrodesis”/exp OR “arthrodesis” OR “ankle arthrodesis” OR “fusion”)	9143

### Study Selection

The abstracts of potential titles were screened for eligibility and 10% double-screened. Two reviewers subsequently performed full-text screening of potentially relevant publications with 10% of papers being double-reviewed. Disagreements were resolved by discussion with a third reviewer, and only publications with TAAs compared with AAs are included. All screening was done in DistillerSR. Citations were tracked in EndNote (Clarivate).

### Data Synthesis

We summarized eligible studies in a narrative form including study design, study objective, funding source, sample size, populations (diagnosis, age, sex), geographic location, interventions, and study quality. The volume of TAA and AA procedures was organized annually starting from the earliest feasible year.

## Results

**Figure 1:**
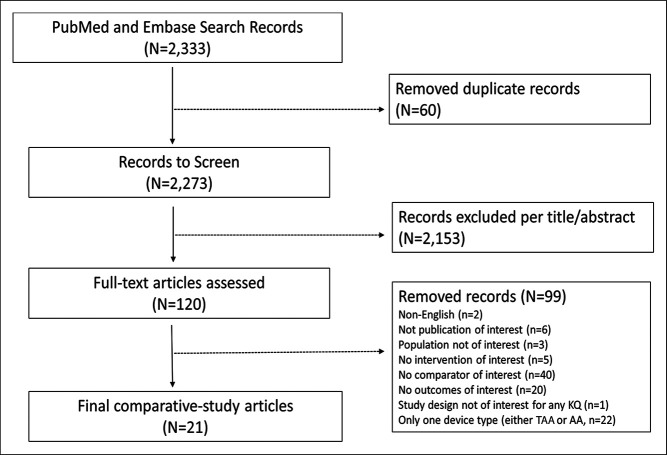
Flow chart detailing systematic selection of included publications comparing utilization trends of total ankle arthroplasty and ankle arthrodesis.

**Figure 2:**
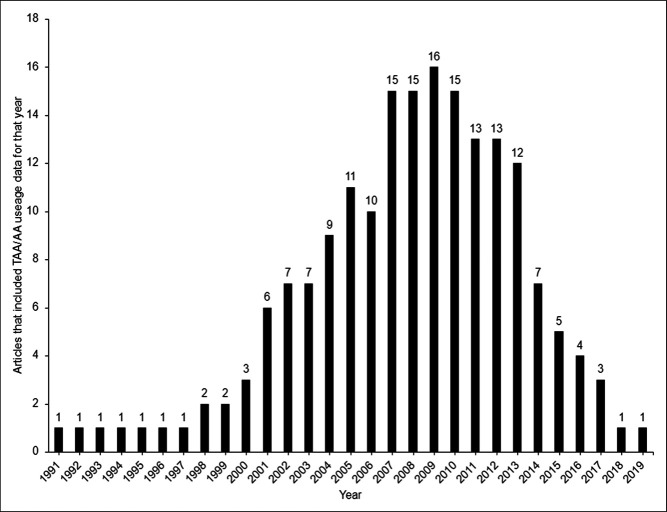
Number of available publications that include usage data comparing total ankle arthroplasty and ankle arthrodesis utilization, over time.

After screening, 21 publications were included in the review (Figure 1). Nine studies originated from the United States and 12 studies from outside the United States (OUS), of which six were from Canada (Table 2). Eight of the nine US-based studies derived data from four national large databases: PearlDiver, National Inpatient Sample, The Medicare Provider Analysis and Review database, and New York Statewide Planning and Research Cooperative System. As for the 12 OUS studies, national databases were used for Canada,^4,5^ Japan,^6^ and Germany^7^ in four studies. The number of publications comparing TAA and AA utilization declined sharply after 2013 (Figure 2).

**Table 2:** Brief Characteristics of all (N = 21) Included Comparative Studies Comparing Total Ankle Arthroplasties and Ankle Arthrodesis

First Author	Year	Country	Usage Years	Usage Presented	Title
Tucker	2020	US	2005-2014	Annually	Nationwide analysis of total ankle replacement and ankle arthrodesis in Medicare patients
Vakhshori	2019	US	2007-2013	Annually	Patient and practice trends in total ankle replacement and tibiotalar arthrodesis in the US from 2007 to 2013
Heckmann	2017	US	2007-2012	Annually	Effect of insurance on rates of total ankle arthroplasty versus arthrodessi for tibiotalar osteoarthritis
Pugely	2014	US	1991-2010	4-year interval	Trends in the use of total ankle replacement and ankle arthrodesis in the US Medicare population
Raikin	2014	US	2000-2010	Total number	Trends in treatment of advanced ankle arthropathy by total ankle replacement and ankle fusion
Terrell	2013	US	2004-2009	Total number	Comparison of practice patterns in total ankle replacement and ankle fusion in the United States
Buza	2017	US	2005 and 2009	Annually	The regionalization of total ankle arthroplasties and ankle fusions in New York State: A 10-year comparative analysis
Ross	2021	US	2010-2019	Total number	Complications following TAA vs. AA for primary ankle osteoarthritis
Norvell	2019	US	2012-2015	Total number	Effectiveness and safety of ankle arthrodesis versus arthroplasty
Matsumoto	2016	Japan	2007-2013	Annually	Time trends and risk factors for perioperative complications in total ankle arthroplasty: r
Dodd	2022	Canada	2001-2013	Total number	Sex differences in end-stage ankle arthritis and following total ankle replacement or ankle arthrodesis
Fischer	2022	Germany	2008-2013	Total number	Superiority of upper ankle arthrodesis over total ankle replacement in the treatment of end-stage posttraumatic ankle arthrosis
Glazebrook	2021	Canada	2007-2017	Total number	Clinical outcome results of total ankle replacement and ankle arthrodesis: a Pilot randomised controlled trial
Desai	2020	Canada	2003-2013	Total number	Role of postoperative patient-reported outcomes to predict ankle arthroplasty and arthrodesis revision
Mehdi	2019	France	2007-2013	Total number	Comparison of 25 ankle arthrodeses and 25 replacements at 67 months' follow-up
Wasik	2019	Poland	2004-2016	Total number	Effect of total ankle arthroplasty and ankle arthrodesis for ankle osteoarthritis: A comparative study
Croft	2017	Canada	2001-2011	Total number	Association of ankle arthritis score with need for revision surgery. Article
Daniels	2014	Canada	2001-2007	Total number	Intermediate-term results of total ankle replacement and ankle arthrodesis: a COFAS multicenter study
Krause	2011	Canada	2002-2007	Total number	Impact of complications in total ankle replacement and ankle arthrodesis analyzed with a validated outcome measurement
Milstrey	2021	Germany	2008-2017	Annually	Trends in arthrodeses and total joint replacements in foot and ankle surgery in Germany during the past decade—Back to the fusion?
Saltzman	2010	Korea	1998-2002	Annually	Treatment of isolated ankle osteoarthritis with arthrodesis or the total ankle replacement: a comparison of early outcomes

**Figure 3:**
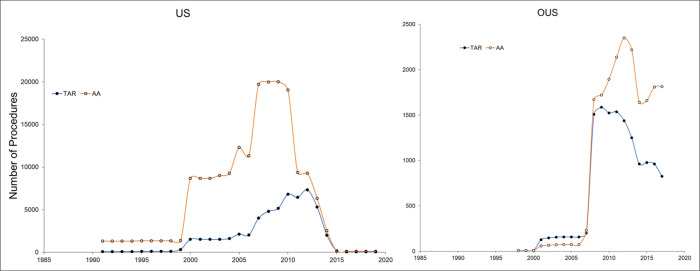
Utilization trends of total ankle arthroplasties and ankle arthrodesis in the United States (left panel) and outside the United States (right panel) as reported in included literature.

The included studies captured between 32,299 TAA patients and 84,411 AA patients in the United States and 1,671 TAA patients and 2,868 AA patients from OUS. These 21 publications captured comparisons between 68,893 TAA and 206,437 AA procedures performed in seven different countries. Although the volume of TAAs reported globally increased from 1998 to 2012, internationally there were mixed trends after 2013 (Figure 3). For example, reported TAA usage in the United States decreased dramatically after 2013. Similarly, France observed a decline in TAA procedures of 3.8% from 2010 to 2019.^8^ By contrast, Korea and Japan reported increased usage of TAA during the same period.^6,9^ Concomitantly, Germany reported a 39.3% decrease and 31.1% increase in the annual number of TAAs and AAs, respectively, from 2008 to 2017.^7^

The number of AA procedures reported in the United States has decreased since a peak in 2009.^10-12^ Unlike the United States, other countries experienced increases in AA during similar periods. Notably, Germany experienced a 31% increase in the annual number of AA procedures from 2008 to 2017,^7^ and Japan saw a general increase in both AA and TAA procedures.^6^

## Discussion

The main finding in this study is that there are different publication trends in terms of TAA and AA utilizations for end-stage ankle OA between different countries and regions. However, there is a decreasing number of publications on the subject, of which 15 of the 21 (71%) included papers in this review were from United States and Canada, skewing the data. The United States has seen a general increase in the number of TAAs reported in publications over the past 2 decades, which coincides with a much smaller increase and general decrease in the number of AA procedures reported during the same period. The number of reported TAA procedures now exceeds the number of AA procedures in the United States. This indicates that the preferred treatment of end-stage OA of the ankle has shifted in the United States from predominantly AA to predominantly TAA.^13^ The longest examined period for percent change, spanning 13 years from 1998 to 2010, found a 546% increase in the annual number of TAAs.^14^ Korea and Japan followed the same upward trend in TAA utilization as the United States.

The decreased usage of TAA in the United States after 2007 was due to a spike in insurance company denial for TAA because of a paper published by Nelson Soo Hoo comparing the incidence of revision after TAA and AA in California.^15^ They found an increased risk of revision after TAA, with 5-year revision approaching 25%, leading to strict criteria for TAA by the insurance companies.

There is conflicting evidence in the literature regarding what constitutes the best surgical treatment for end-stage OA of the ankle.^16^ The shift in preferred treatment of end-stage ankle OA from AA to TAA in some regions is therefore not consensus or science-based and may be driven by industry or patient demand, as arthroplasty maintains movement in the ankle, which may be preferred by the patients.^17^ The publication trend in Europe contrasted the United States, with a general decline in TAA procedures reported in France and Germany after 2013, except in Italy, which had a similar increase in TAA procedures from 2001 to 2016 as the United States.^18^ The decline in TAA usage in Europe may be linked to high revision rates reported from the national registries, particularly in Scandinavia.^19^

The variation in treatment for end-stage ankle OA globally is not surprising because there are no clear guidelines on what constitutes the best surgical treatment.^16^ Until such guidelines exist, the variation is understandable and may even be of benefit to researchers. This variation is an opportunity to learn more about the advantages and disadvantages of the different treatments, but this requires more comparative studies and harmonized data reporting. One major issue is that the national registries keep track of the TAAs, but not the AA procedures. Better postmarket surveillance data, including long-term patient-reported outcome and revision rates, are needed, particularly for arthrodesis procedures.

## Limitations

This is a systematic literature review of comparative studies reporting utilization of TAA and AA. The real-world utilization trends may be different from the studies published, although it is likely that the published literature reflects clinical practice. Furthermore, the lack of routine registration of AA in registries may skew the available data. The retrospective analysis of the literature and considerable variations in the reported periods limit the direct comparison and generalizability of our findings. Because each literature encompasses its own specific patient demographic, making broad generalizations are challenging. Moreover, the identification of patients who underwent TAA or AA was primarily done via the International Classification of Diseases or Current Procedural Terminology codes. Errors or absences in coding could also conceivably influence findings.

## Conclusion

There is limited and declining number of publications describing the utilization trends of TAA and AA procedures for end-stage ankle OA. Publication describing the use of TAA procedures has generally increased in the United States and some Asian countries, whereas publications describing AA has declined. In Europe, the publication trend of TAA devices is generally decreasing and AA has increased, except in Italy. Further studies are needed to describe and evaluate the discrepancy in surgical treatment methods for end-stage ankle OA across nations.
